# Genotypic variation in water-use traits and drought response of Rwandan common bean cultivars

**DOI:** 10.1093/aob/mcag257

**Published:** 2026-08-17

**Authors:** Diana Laura Labastida Jaimes, Jules Maurice Shema, Jean-Pierre Muhire, Donat Nsabimana, Karin S L Johansson, Mats X Andersson

**Affiliations:** Department of Biological and Environmental Sciences, University of Gothenburg, SE-405 30 Gothenburg, Sweden; Department of Biological and Environmental Sciences, University of Gothenburg, SE-405 30 Gothenburg, Sweden; School of Forestry and Biodiversity Conservation, College of Agriculture, Animal Sciences and Veterinary Medicine, University of Rwanda, PO Box 210, Musanze, Rwanda; Rwanda Agriculture and Animal Resources Development Board, Musanze Station, PO Box 5016, Huye, Rwanda; School of Forestry and Biodiversity Conservation, College of Agriculture, Animal Sciences and Veterinary Medicine, University of Rwanda, PO Box 210, Musanze, Rwanda; Department of Biological and Environmental Sciences, University of Gothenburg, SE-405 30 Gothenburg, Sweden; Department of Biological and Environmental Sciences, University of Gothenburg, SE-405 30 Gothenburg, Sweden

**Keywords:** *Phaseolus vulgaris*, water-use efficiency, common bean, drought, gas exchange, stomata, genetic variation

## Abstract

**Background and Aims:**

Water availability is the most limiting factor for global agricultural productivity. Rain-fed smallholder farming common to sub-Saharan Africa is particularly sensitive to unpredictable precipitation patterns and periods of drought – issues that are projected to be exacerbated by ongoing climate change. Common bean (*Phaseolus vulgaris*) is widely cultivated in East Africa and represents an important source of protein, carbohydrates, micronutrients and income for smallholder agriculture. Bean water-use traits and productivity responses to water limitation are thus of great importance. In this study we sought to investigate the genetic variation of water-use traits and responses to terminal drought among Rwandan landraces and improved cultivars of common bean.

**Methods:**

Eighteen landraces and 12 improved cultivars were cultivated under realistic field conditions and evaluated for physiological traits related to water use, phenological properties and effects of terminal drought treatment.

**Key Results:**

Considerable genetic variation was detected across landraces and improved cultivars for all investigated traits. There was a strong negative correlation between yield and intrinsic water-use efficiency. High yielding cultivars demonstrated low water-use efficiency. There was, however, also notable variation in this relationship, with some cultivars combining water-conserving properties and high productivity. Phenological data indicated a positive relationship between productivity and time to maturity. The lack of a consistent association between water-use traits and drought responses demonstrates that traits for drought avoidance and tolerance are genetically distinct and regulated separately.

**Conclusions:**

Our results demonstrate a large degree of genetic variation and highlight the possibility of combining traits for water economy and drought tolerance while maintaining yield in common bean. The contrasting genotypes reported in this study could be used for genetic mapping of genomic regions, and cultivars combining high grain yield with moderate water use and moderate tolerance to drought are suggested for future breeding efforts.

## INTRODUCTION

Climate change effects on global agricultural productivity and food security are already apparent and projected to be further exacerbated under all emission scenarios (IPCC, 2022). Crops are exposed to multiple climate-related stressors that reduce overall yields and yield stability. Global warming increases atmospheric evaporative demand, resulting in increased water requirements in crop production (IPCC, 2021, 2022; López *et al*., 2021). In addition, extreme weather events are becoming more frequent. For example, periods of drought are becoming longer and more unpredictable (IPCC, 2021). Irrigation and other high-input agricultural practices may provide short-term alleviation but will in many cases have long-term environmental consequences such as freshwater depletion and soil degradation (Rosa *et al*., 2020; IPCC, 2022; Abdo *et al*., 2025). Improving crop water economy and drought resilience is thus critical for sustaining agricultural productivity in the face of global change (Atlin *et al*., 2017).

Common bean (*Phaseolus vulgaris*) is the most important grain legume for direct human consumption (Broughton *et al*., 2003; Uebersax, 2023) and constitutes a low-input, sustainable source of protein and micronutrients such as iron and zinc (Foyer *et al*., 2016; IPCC, 2022). Populations in large parts of Africa, Latin America and Asia rely on common bean as a major source of dietary protein (Assefa *et al*., 2019). In Rwanda, per-capita bean consumption is the highest in the world, with households consuming beans on average 6 d per week (National Early Child Development Program and World Food Programme, 2018; Assefa *et al*., 2019). While substantial breeding efforts have targeted productivity and yield stability in major cereal crops, there has been considerably less focus on the improvement of legumes (Larochelle and Alwang, 2022). In fact, the increase in legume production has historically been associated mostly with expansion of cultivated area (Foyer *et al*., 2016). There is thus much potential for improving the productivity of beans, particularly under unfavourable environmental conditions (Beebe *et al*., 2008; Hu *et al*., 2025) and a smallholder context.

Landraces and wild relatives often provide lower yields compared to modern crop varieties but may harbour more genetic variation in abiotic stress tolerance (Beebe *et al*., 2013; Dwivedi *et al*., 2016; Gilliham *et al*., 2017; Tehseen *et al*., 2022; del-Canto *et al*., 2025). Rwanda is a secondary centre of common bean diversity and is known for a wide variety of landraces (Sperling, 2001; Blair *et al*., 2010; Chikasha *et al*., 2026). The traditional practice of growing seed mixtures rather than single varieties and consumer tolerance of a wide range of seed types maintains a high level of diversity in cultivation. However, adoption of improved varieties may decrease the actual cultivated diversity present. The potential for using the Rwandan common bean diversity as a genetic resource for improved abiotic stress tolerance has, to our knowledge, not been previously investigated. Furthermore, the climbing varieties that are increasingly promoted in Rwanda due to their higher productivity have received considerably less attention by the scientific community than bush varieties, which are more suitable for mechanical harvest (Beebe *et al*., 2013; Katungi *et al*., 2018).

Plants cope with dry conditions through escape, avoidance or tolerance strategies (Ilyas *et al*., 2021). Escape is mediated by accelerated development in response to drought stress. Avoidance mechanisms, such as deeper root systems, conservative water use and stomatal responsiveness, maintain tissue water potential despite limited water availability. Tolerance is mediated by traits such as high osmolyte concentrations and cell wall elasticity, which enable turgor pressure to be maintained at lower water potentials. Yield under dry conditions is a complex trait affected by numerous underlying physiological parameters, often representing several of the above strategies, as well as by variability in environmental conditions between years or sites (Richards *et al*., 2002). Heritability is therefore often low, making the identification of favourable genetic variants challenging. The underlying physiological traits, on the other hand, tend to be more genetically stable (Rebetzke *et al*., 2002; Richards, 2006). In addition, physiological traits that can be assessed in early life stages are important for the implementation of rapid breeding cycles (Richards, 2006; Dembele *et al*., 2025).

Although some recent progress has been made regarding the development of stress-tolerant common bean varieties, there is a strong need for more knowledge about the genetic variation in traits related to abiotic stress resilience (Beebe *et al*., 2013; Gilliham *et al*., 2017). Previous breeding efforts for drought resilience in common bean have focused on traits related to the following broad categories: early maturity, accelerated development or partitioning to seeds in response to stress; overall yield improvement, which may result in improved yield under drought despite large decreases; and drought avoidance by improvement of root traits. Disease resistance, particularly to soil-borne diseases that exacerbate drought impacts on roots, has also improved yields under dry conditions (Beebe *et al*., 2013). Water economy has received relatively less attention, despite a large potential for improvement in legumes (Adams *et al*., 2018; Sanz-Saez *et al*., 2019; Buckley *et al*., 2025).

Intrinsic water-use efficiency (iWUE), i.e. the ratio between photosynthetic carbon fixation (*A*_net_) and stomatal conductance (*g*_s_), is a promising target trait for improvement of crop water economy. This has been demonstrated by the successful development of wheat cultivars with up to 40 % higher yields under dry conditions (Rebetzke *et al*., 2002). Stable carbon isotope signatures (δ^13^C) of leaf tissue samples can be used in C3 plants as a high-throughput estimate of iWUE. The extent of discrimination against ^13^C in carbon assimilation is determined by the availability of CO_2_ inside the leaf, which in turn is affected by *A*_net_ and *g*_s_ (Farquhar *et al*., 1989; Condon *et al*., 2002). Leaf δ^13^C provides a time-integrated estimate of iWUE that covers the entire period of leaf formation, as opposed to point estimates obtained from leaf gas exchange measurements. However, as the analysis of iWUE derived from δ^13^C does not allow for separation of the underlying effects of *A*_net_ and *g*_s_ on iWUE, it is preferable to combine δ^13^C estimates and leaf gas exchange measurements in studies of variation in iWUE (Condon *et al*., 2002).

In this study, we assessed the variation of an array of physiological traits related to drought resilience in a panel of 30 common bean genotypes from Rwanda. These genotypes represent landraces collected from regions with a large range in climatic conditions, and improved varieties released within the Rwandan bean breeding programme. We quantified phenological traits related to drought escape, water economy traits related to drought avoidance, and traits related to drought tolerance and yield under terminal drought stress. To our knowledge, this is the first study of physiological traits related to drought resilience in Rwandan cultivars of common bean.

## MATERIAL AND METHODS

### Plant material and cultivation

The study was conducted using 30 Rwandan common bean (*Phaseolus vulgaris* L.) cultivars with climbing growth habit. Eighteen landraces (Table 1), collected in the 1980s, were provided by the International Center for Tropical Agriculture (CIAT), and 12 improved cultivars (Table 2) released from the Rwandan bean breeding programme were provided by the Rwanda Agriculture and Animal Resources Development Board (RAB). Landraces were selected to represent a broad geographical and climatic diversity, to represent both Andean and Mesoamerican gene pools (as inferred from seed phaseolin type information in the CIAT genebank database, Table 1) as well as contrasting phenotypes for key traits of interest as observed in pilot experiments. The improved cultivars (Table 2) are used in breeding and cultivation across Rwanda.

**Table 1.** Landraces selected for field experiment in Rwanda.

Accession	Synonym	Year of collection	Gene pool (inferred from seed protein)	Climate type	Rainfall regime	Key features at origin
**G20737**	BYUMBA 9	1986	Andean	Temperate	Sub-humid	Mid-elevation region with annual temperatures (∼20 °C), and seasonal rainfall (∼900 mm year^−1^). The site experiences a pronounced wet season and a dry period during mid-year
**G20849B**	KIBUNGO 10	1986	Mesoamerican	Temperate	Sub-humid	Mid-elevation site (∼1400–1600 m). Annual temperature ∼20 °C and annual rainfall ∼900 mm. Rainfall is highly seasonal, with a pronounced wet season (March–May) and a short dry period (June–August)
**G20785**	GIKONGORO 10	1986	Mesoamerican	Temperate	Humid (seasonally wet)	Highland to mid-elevation area (∼1600–1800 m) with mean annual temperature ∼19 °C. Rainfall exceeding 1100 mm. Rainfall is highly seasonal, with intense precipitation from March to May and a brief dry period around mid-year
**G20898**	KIBUYE 8	1986	Andean	Temperate–cool	Humid	Highland area (∼1700–1900 m) with mean annual temperature ∼18 °C and annual rainfall >1200 mm. Rainfall is seasonal, with two wet periods (March–May and October–December) and a short dry season in mid-year.
**G20760**	CYANGUGU 12	1986	Andean	Temperate–cool	Humid	Highland area (∼1600–1900 m) with mean annual temperature ∼19 °C and annual rainfall >1400 mm. Rainfall is strongly seasonal, with two wet periods (March–May and October–December) separated by a short mid-year dry season.
**G21718**	PRODUCTIVO 2	1983	Andean	Temperate	Humid	Mid- to high-elevation area (∼1700–1900 m) with mean annual temperature ∼20 °C and annual rainfall ∼1280 mm. Rainfall is moderately seasonal, with two wet periods (March–May and October–December) and a short dry interval in mid-year.
**G20782**	GIKONGORO 7	1986	Mesoamerican	Temperate	Humid	Highland area (∼1700–1900 m) with mean annual temperature ∼19 °C and annual rainfall >1300 mm. Rainfall is strongly seasonal, with peaks from March to May and again from October to December, separated by a brief dry period mid-year.
**G20796**	GISENYI 4	1986	Andean	Temperate	Humid	Mid- to high-elevation area (∼1600–1800 m) with mean annual temperature ∼20 °C and annual rainfall ∼1250 mm. Rainfall is moderately seasonal, with two pronounced wet periods (March–May and October–December) separated by a brief dry season around mid-year.
**G20736**	BYUMBA 8	1986	Mesoamerican	Temperate	Sub-humid	Mid-elevation area (∼1500–1700 m) with mean annual temperature ∼19 °C and annual rainfall ∼900 mm. Rainfall is strongly seasonal, with a wet season from March to May and a pronounced dry period during June–August.
**G20734**	BYUMBA 6	1986	Mesoamerican	Temperate	Sub-humid	Mid-elevation area (∼1400–1600 m) with mean annual temperature ∼19 °C and annual rainfall ∼980 mm. Rainfall is seasonal, with a distinct dry period during mid-year and two wet seasons (March–May and October–December).
**G20973**	RUHENGERI 30	1986	Andean	Cool	Humid	Highland area (>1900 m) with mean annual temperature ∼17 °C and annual rainfall ∼1300 mm. Rainfall is moderately seasonal, with two wet periods (March–May and October–December) separated by a short dry season in mid-year.
**G20833**	GITARAMA 16	1986	Mesoamerican	Temperate	Humid	Mid- to high-elevation area (∼1600–1800 m) with mean annual temperature ∼18 °C and annual rainfall >1200 mm. Rainfall is highly seasonal, with wet peaks in March–May and October–December, separated by a brief dry period mid-year.
**G20749**	CYANGUGU 1	1986	Mesoamerican	Temperate	Humid	Mid-elevation area (∼1500–1700 m) with mean annual temperature ∼19 °C and annual rainfall ∼1400 mm. Rainfall is highly seasonal, with two wet periods (March–May and October–December) separated by a short dry season mid-year.
**G20799A**	GISENYI 7	1986	Andean	Temperate	Sub-humid	Mid-elevation area (∼1500–1700 m) with mean annual temperature ∼21 °C and annual rainfall ∼930 mm. Rainfall is strongly seasonal, with wet peaks in March–May and October–December, separated by a marked dry period mid-year.
**G20830**	GITARAMA 13	1986	Mesoamerican	Temperate	Humid (seasonally wet)	Mid-elevation area (∼1500–1700 m) with mean annual temperature ∼20 °C and annual rainfall ∼1150 mm. Rainfall is highly seasonal, with wet peaks from March to May and October to December, and a distinct dry period around June–August.
**G20801**	GISENYI 9	1986	Andean	Temperate	Humid	Mid- to high-elevation area (∼1600–1800 m) with mean annual temperature ∼19 °C and annual rainfall ∼1280 mm. Rainfall is highly seasonal, with two main wet periods (March–May and October–December) and a brief dry season during mid-year.
**G20874**	KIBUNGO 35	1986	Andean	Temperate warm	Sub-humid	Mid- to low-elevation area (∼1400–1600 m) with mean annual temperature ∼20.5 °C and annual rainfall ∼880 mm. Rainfall is strongly seasonal, with wet periods from March to May and October to December, separated by a pronounced dry season in mid-year.
**G20758**	CYANGUGU 10	1986	Mesoamerican	Warm	Sub-humid to humid	Low- to mid-elevation area (∼1200–1400 m) with mean annual temperature ∼22 °C and annual rainfall ∼1200 mm. Rainfall is seasonal, with wet periods from March to May and October to December, separated by a short dry season in mid-year.

**Table 2.** Improved cultivars of common beans selected for field experiment.

Release name	Pedigree	Origin	Adaptation to altitude	Abiotic stress tolerance traits	Biotic stress tolerance traits
**ISAR CB 103**	Gasilida (Landrace)	Local collection	High–medium	Low soil fertility	Angular leaf spot, ascochyta blight, anthracnose, bean common mosaic virus
**ISAR CB 107**	MAC 44	CIAT	Medium–low	Low soil fertility, drought tolerant	Angular leaf spot, ascochyta blight, bean common mosaic virus
**ISAR CB 1046**	MAC 9	CIAT	Medium–low	Drought tolerant	Angular leaf spot, ascochyta blight, anthracnose, bean common mosaic virus
**RAB CB 122**	RWV 2872	Rwanda	High–medium	Heavy rain	Angular leaf spot, ascochyta blight, anthracnose, bean common mosaic virus
**RAB CB 123**	RWV 3316	RAB	High		Angular leaf spot, ascochyta blight, anthracnose, bean common mosaic virus
**RWIBARURA**	RWIBARURA		High		Anthracnose, ascochyta blight, bacterial blight, halo blight, fusarium, pythium, rhizoctonia, rust
**RW CB 186**	RWV 2850 A-2B		All (low–high)		Anthracnose, ascochyta blight, bean common mosaic necrosis virus, bacterial blight, halo blight, fusarium, pythium, rhizoctonia, rust
**ISAR CB 102**	RWV 1129	ISAR	High–medium	Heavy rain	Angular leaf spot, ascochyta blight, anthracnose, bean common mosaic virus
**ISAR CB 104**	CAB 2	CIAT	High	Heavy rain, low soil fertility	Angular leaf spot, ascochyta blight, anthracnose, bean common mosaic virus
**RAB CB 121**	RWV 3006	RAB	High–medium		Angular leaf spot, ascochyta blight, anthracnose, bean common mosaic virus
**RAB CB 124**	RWV 3317	RAB	High		Angular leaf spot, ascochyta blight, anthracnose, bean common mosaic virus
**RAB CB 128**	RWV 2887	RAB	High		Angular leaf spot, ascochyta blight, anthracnose, bean common mosaic virus

Field experiments were conducted during two dry seasons (July–November 2022 and May–October 2023) at the RAB Rubona research station in Huye, Rwanda (2°28′55.5″S, 29°45′58.5″E). This location represents intermediate conditions in Rwanda in terms of altitude, temperature and precipitation and is located at 1600 m a.s.l. (Tarvainen *et al*., 2022). The field was used for potato prior to the 2022 experiment and for upland rice prior to the 2023 experiment. Both experiments were designed to expose plants to terminal drought during the pod-filling stage. The soil at the site is classified as sandy clay to sandy clay loam, with moderate to high clay content, moderate acidity, and low to moderate content of organic matter and nitrogen (Tarvainen *et al*., 2022).

Before sowing, the field was prepared by manual tillage, weeding and application of cattle manure (∼20 000 kg ha^−1^) followed by diammonium phosphate (53 kg ha^−1^). Urea (53 kg ha^−1^) was top-dressed during weeding 2–3 weeks after sowing. Staking with one stake per plant was conducted when the seedlings were established and started to need support. During the well-watered phase of the experiment, irrigation was applied manually using garden hoses with spray lances (Premium Spray Lance 90 cm, Gardena). Water was sourced from a nearby river and temporarily stored in an irrigation dam. Flow-through meters (AquaCount, Gardena) were used at the beginning of the experiment to verify that the amount of water delivered (∼500–600 L of water per plot) approximated normal rainfall at the site during the growing season (Dusenge *et al*., 2021). Thereafter, similar volumes were applied. Irrigation was skipped on rainy days. Some interruptions in the irrigation regime occurred during the first few weeks of 2022 due to water supply issues. Soil surface loosening was performed periodically to improve infiltration (four times in 2022 and six times in 2023). Aphid infestations were controlled with Rocket (Sunrise Agro Industries, India) applied four times in 2022 and twice in 2023, with irrigation suspended on application days.

### Experimental design

Eight plots (8 × 10 m) were established (Supplementary Data Fig. S1). Within each plot, cultivars were planted completely randomized with five replicates each. Plots contained eight rows with an inter-row space of 1 m and 0.5 m between plants within a row, totalling ∼150 plants per plot. Plots were separated by 2-m buffer zones (Fig. S1). Irrigation was maintained in all plots until pod filling (∼60 d after sowing). Drought was imposed by reducing irrigation in every second plot: daily irrigation was reduced to every 3 d for 1 week and then completely stopped 67 d after sowing. Control plots remained irrigated throughout the experiment.

### Meteorological and soil moisture measurements

Air temperature and relative humidity were recorded using three data loggers (Tinytag Plus 2, Gemini, UK) positioned 1.5 m above ground at regular intervals along the field and protected from direct sunlight by aluminium covers. Data were recorded every 10 min in 2022 and as this frequent sampling was deemed unnecessary it was logged every 30 min in 2023. Soil volumetric water content was quantified twice per week at depths of 100, 200, 300 and 400 mm using a PR2 profile probe with two access tubes installed in each plot (Delta-T Devices Ltd, UK).

### Phenology, plant health and yield

Phenological traits recorded included days to flowering (first open flower) and days to maturity (when 50 % of the pods lost pigmentation and began to dry). Plant overall health status was estimated using vigour scores on a 1–9 scale, where 1 represents ‘excellent’ and 9 ‘very poor’ (van Schoonhoven and Pastor-Corrales, 1987). At harvest, pod number, seed number, total pod weight and total seed weight per plant were determined. Specific seed weight was calculated as the total seed weight divided by the number of seeds per plant. Pod harvest index was calculated as the ratio of seed weight to the total pod weight.

### Leaf-level physiological measurements

Leaf gas exchange was measured in 2022 on young unstressed plants 35–47 d after sowing. Two to three within-plot replicates per cultivar were measured. Net CO_2_ assimilation (*A*_net_) and stomatal conductance (*g*_s_) were measured on a healthy, fully expanded, sun-exposed trifoliate leaf using an LI-6800 portable photosynthesis system (LI-COR Biosciences, USA) between 0900 and 1330 h on clear days. Each measurement lasted approximately 2 min per plant. The measurements were survey measurements intended to capture a snapshot of the state of the leaves prior to being enclosed in the measurement chamber, which is achieved by keeping the conditions inside the chamber as similar to ambient as possible and allowing for the chamber, but not the leaf, to equilibrate. Hence, *g*_s_ should not change from the prior condition. Chamber reference conditions were kept as constant as possible throughout the measurements, with a CO_2_ concentration set to 420 µmol mol^−1^ and a photosynthetic photon flux density (PPFD) of 1800 µmol m^−2^ s^−1^, which is within the range of full sunlight under field conditions. The temperature exchanger was set to match the value of ambient air temperature, which was measured every 30 min in the shade. Relative humidity was not actively controlled but was limited when required to avoid condensation inside the instrument. SPAD index as a proxy for chlorophyll content (Uddling *et al*., 2007) was measured using a SPAD-502 meter (Konica Minolta, Japan) on the same leaf.

Leaf carbon stable isotope composition (δ^13^C) as a proxy for iWUE was determined from a healthy, fully expanded trifoliate leaf collected after completion of the gas exchange measurement campaign. Samples were dried at 70 °C for at least 48 h, ground to a fine powder in a Mixer Mill MM 400 (Retsch, Germany), and ∼5 mg was weighed into tin capsules using a Mettler AT20 balance (Mettler Toledo). Samples from 2022 were analysed on an elemental analyser (ANCA-GSL; Sercon Ltd, UK) coupled to an isotope ratio mass spectrometer (20-22 IRMS; Sercon Ltd) at the ISOGOT facility (Department of Earth Sciences, University of Gothenburg). Samples from 2023 were analysed using a similar setup (ANCA-GSL coupled to a 20-20 IRMS; Sercon Ltd) at the UC Davis Stable Isotope Facility (Davis, CA, USA). δ^13^C values were expressed relative to the VPDB (Vienna Pee Dee Belemnite) standard.

iWUE was estimated from leaf carbon isotope composition (δ^13^C) following the model of Farquhar *et al*. (1989). Photosynthetic carbon isotope discrimination (Δ^13^C) was first calculated as: Δ^13^C = (δ*a* − δ*p*)/(1 + δ*p*/1000) where δ*a* is the isotopic composition of atmospheric CO_2_ and δ*p* is the isotopic composition of plant tissue. Atmospheric δ^13^C values for 2022 and 2023 were obtained from the Mauna Loa Observatory record of the Scripps CO_2_ Program (Scripps CO_2_ Program, 2024). The ratio of intercellular to atmospheric CO_2_ concentration (*C*_i_/*C*_a_) was then estimated as: *C*_i_/*C*_a_ = (Δ^13^C − *a*)/(*b* − *a*) where *a* = 4.4‰ represents isotopic fractionation during diffusion through stomata and *b* = 27‰ represents fractionation during carboxylation. Finally, iWUE was calculated as: iWUE = [*C*_a_(1 − *C*_i_/*C*_a_)]/1.6.

Leaf osmotic potential was quantified in leaf samples collected pre-dawn, following Bartlett *et al*. (2012*a*) as modified by Wittemann *et al*. (2024). Leaf discs (10 mm) sampled from a healthy, fully expanded trifoliate leaf (avoiding the central vein) were flash-frozen in liquid nitrogen, transported in a cryoshipper and stored at −80 °C until analysis. A smaller disc (5 mm) was then subsampled and analysed using a VAPRO 5600 vapour pressure osmometer (EliTechGroup, France). Upon thawing, the discs were punctured 8–10 times with tweezers and in no more than 15 s transferred to the osmometer. Osmolality was measured every 2 min until values stabilized.

### Statistical analyses

All statistical analyses were conducted in R (v.4.4.1; R Core Team, 2024) using a significance level of α = 0.05. Variation in leaf-level physiological traits (gas exchange parameters, SPAD, osmotic potential and iWUE derived from δ^13^C) among cultivars was analysed using one-way ANOVA. Differences in iWUE between improved cultivars and landraces were assessed using Welch’s two-sample *t*-test. Relationships among physiological traits and yield parameters were assessed using correlation analysis.

The effects of drought, genotype and their interaction on agronomic traits (yield per plant, pods per plant, number of seeds per plant and specific seed weight) were analysed separately for each year (2022 and 2023) using generalized linear mixed models (GLMMs) fitted with a Tweedie error distribution and a log link function, as implemented in the glmmTMB package. The Tweedie distribution was selected because yield is continuous, non-negative, and includes zero or near-zero values. Genotype, treatment (irrigation vs. drought) and their interaction were included as fixed effects, while plot was included as a random intercept to account for spatial variation. Separate models were fitted for each year.

Estimated marginal means (EMMs) and treatment contrasts were obtained using the emmeans package. EMMs represent model-adjusted means derived from the fitted GLMMs, allowing comparisons among genotypes and irrigation treatments after accounting for the effects included in the model. Treatment contrasts were used to estimate the effect of drought relative to irrigation for each genotype. Yield responses are presented as drought-to-irrigation ratios with 95 % confidence intervals. A significant drought effect was inferred when the confidence interval did not overlap zero.

Model diagnostics were conducted using the DHARMa and performance packages.

## RESULTS

### Environmental conditions and drought treatment

Field trials using a selection of landraces (Table 1) and improved cultivars (Table 2) of common bean from Rwanda were conducted during two consecutive dry seasons (May to September). Average temperature during the day in both years varied from 14.7 to 28.3 °C with occasional peaks reaching up to 36.8 °C in the afternoon. Mean daytime temperature in 2022 was 23.6 °C and in 2023 was 24.9 °C (Supplementary Data Fig. S2). Relative humidity fluctuated in both years, ranging from 32 to 90 % in 2022 and from 36 to 100 % in 2023 (Fig. S2). The soil moisture content was maintained through irrigation until the pod filling stage when irrigation was decreased and eventually suspended for drought-treated plots (Fig. 1). Soil volumetric water content showed a clear decline following drought initiation (Fig. 1). In 2022, initial problems with water supply and unexpected rain towards the end of the experiment caused more irregular soil moisture patterns, whereas in 2023 conditions were more stable. Despite these variations, the drought treatment reduced the volumetric soil water content at 40 cm depth from above 20 % to below 10 % during the pod-filling stage.

**Fig. 1.**
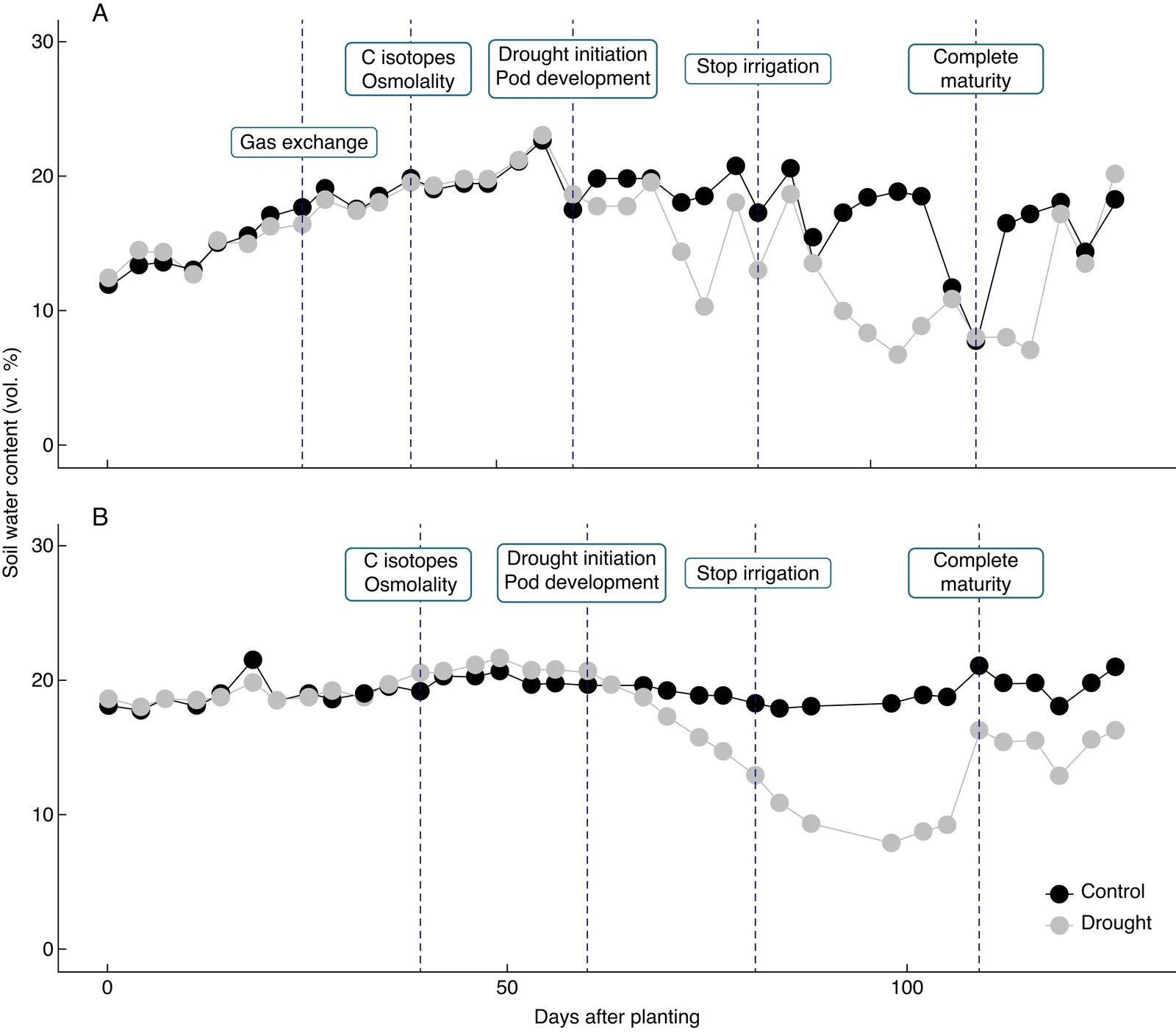
Soil volumetric water content and experimental events during the two field seasons in Rwanda. The average soil volumetric water content (vol.%) in irrigated (black) and drought-treated (grey) plots measured twice weekly at 0–40 cm depth throughout the field seasons 2022 (A) and 2023 (B). Vertical dashed lines indicate the timing of key sampling or developmental stages: gas exchange measurements, leaf sampling for δ^13^C and osmotic potential, drought-reduced irrigation/pod development, termination of irrigation and when all plants had reached full maturity.

### Genotypic variation in leaf physiological traits

Gas exchange measurements, and leaf sampling for carbon isotope ratios used to estimate iWUE and for osmolality were performed on unstressed plants prior to drought treatment as indicated in Fig. 1. Although some plants started flowering during the end of the measurement campaign, most plants were in a vegetative stage. The two landraces *RWIBA* and *KIB_8* were excluded from the statistical analyses of the leaf-level physiological traits due to poor germination and performance, which resulted in an insufficient number of replicate plants that had healthy leaves at the time of measurements and sampling for leaf-level traits. One-way ANOVA showed significant differences among genotypes for net photosynthesis (*A*_net_; *F*_(29,494)_ = 3.92, *P* < 0.001; *n* = 20; Fig. 2A) and stomatal conductance (*g*_s_; *F*_(29,494)_ = 3.13, *P* < 0.001; *n* = 20; Fig. 2B), indicating substantial genetic variation in photosynthetic and stomatal regulation. When averaged by genotype, mean *A*_net_ varied approximately 1.7-fold between the lowest- and highest-performing genotypes, ranging from 14.9 µmol m^−2^ s^−1^ (KIB_35) to 25.8 µmol m^−2^ s^−1^ (ISAR_102). Leaf chlorophyll content (SPAD) also differed significantly among genotypes (*F*_(28,449)_ = 3.90, *P* < 0.001; *n* = 20; Fig. 2C). iWUE derived from δ^13^C exhibited significant genotypic variation among the tested cultivars (*F*_(29,439)_ = 3.19, *P* < 0.001; *n* = 8; Fig. 3). Landraces displayed significantly higher iWUE than improved cultivars (Welch’s *t*-test *t*_(401.6)_ = −3.88, *P* < 0.001, improved *n* = 160, landrace *n* = 240), with mean values of 98.5 and 93.9 μmol mol^−1^, respectively, although substantial overlap between groups was observed. A significant effect of year was detected, with cultivars exhibiting higher iWUE in 2023 than in 2022 (paired *t*-test; mean difference = −5.76 μmol mol^−1^, 95 % CI = −7.47 to −4.05; *P* < 0.001). Despite this interannual variation, iWUE was strongly correlated across years (Spearman’s ρ = 0.56, *P* = 0.002), indicating a stable genotypic ranking for this trait.

**Fig. 2.**
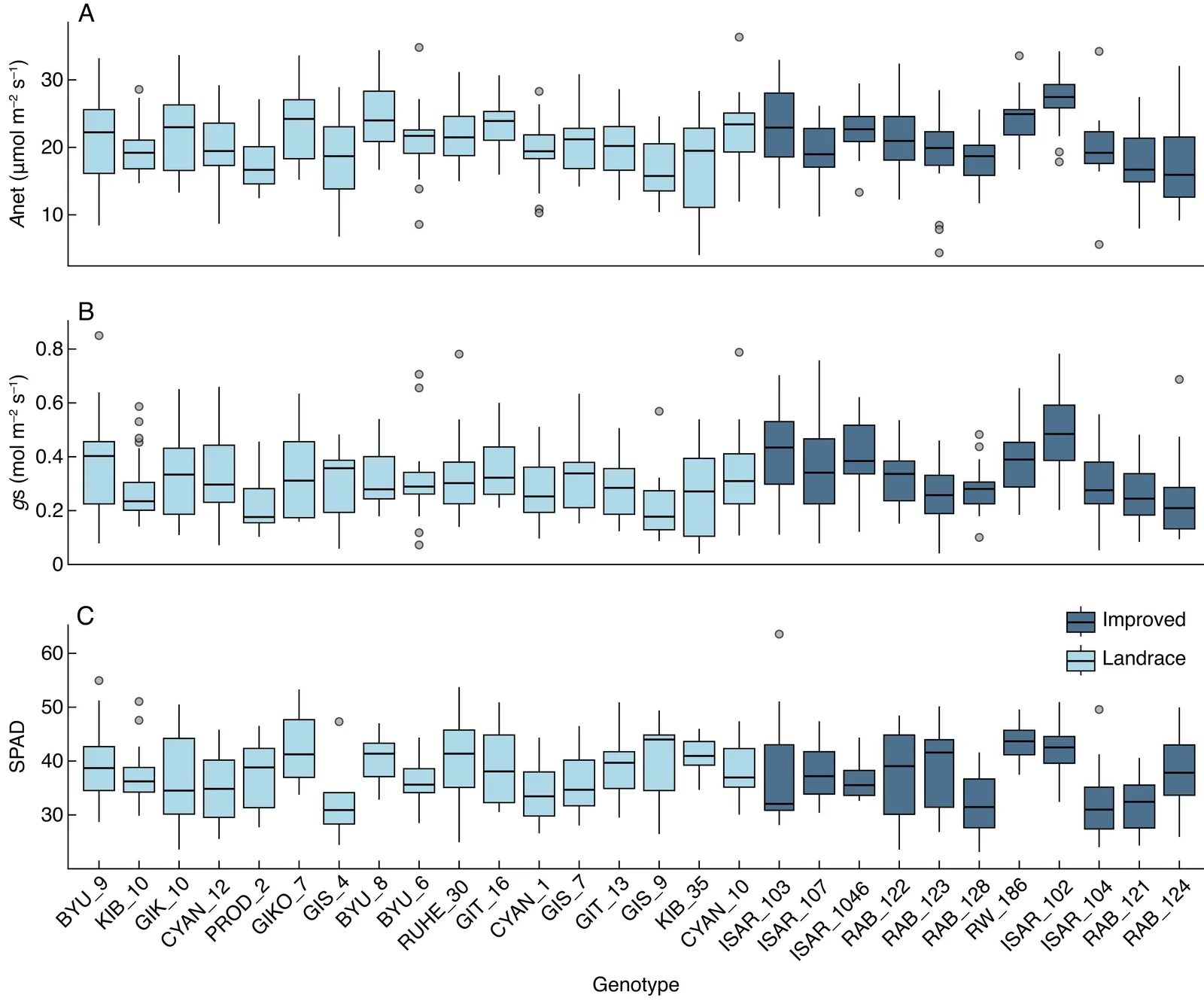
Leaf-level physiological traits. Photosynthetic rate (*A*_net_, A), stomatal conductance (*g*_s_, B) and chlorophyll content (SPAD, C) were measured in bean landraces and improved cultivars on a healthy, sun-exposed, fully expanded trifoliate leaf under irrigated conditions. Light blue boxes represent landraces, and dark blue boxes represent improved cultivars. Boxplots show interquartile range (boxes), median (horizontal line) and individual outliers as dots.

**Fig. 3.**
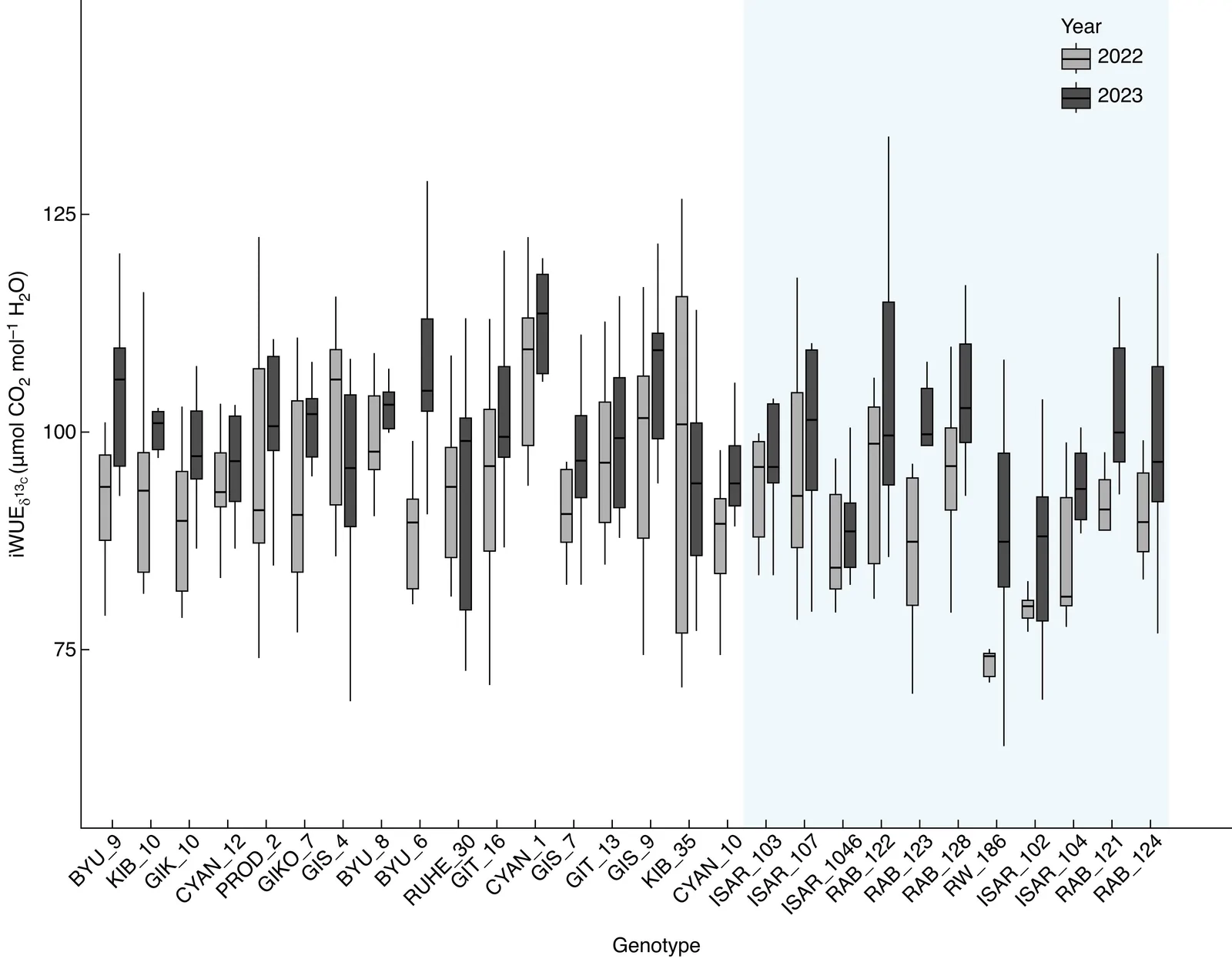
Intrinsic water use efficiency determined by leaf carbon isotope discrimination. Intrinsic water-use efficiency (iWUE, µmol CO_2_ mol^−1^ H_2_O) estimated from leaf carbon isotope composition (δ^13^C) in irrigated plants across two field seasons in Rwanda. Boxplots show interquartile range (boxes), median (horizontal line) and individual outliers as dots. Light grey: 2022; dark grey: 2023. The light blue shading corresponds to improved cultivars.

Leaf osmolality varied significantly among genotypes (*F*_(29,394)_ = 4.56, *P* < 0.001) and between years (*F*_(1394)_ = 141.66, *P* < 0.001), with no significant genotype × year interaction (*P* = 0.052; Fig. 4). Leaf osmolality was consistently higher in 2022 than in 2023, with no systematic difference between landraces and improved cultivars.

**Fig. 4.**
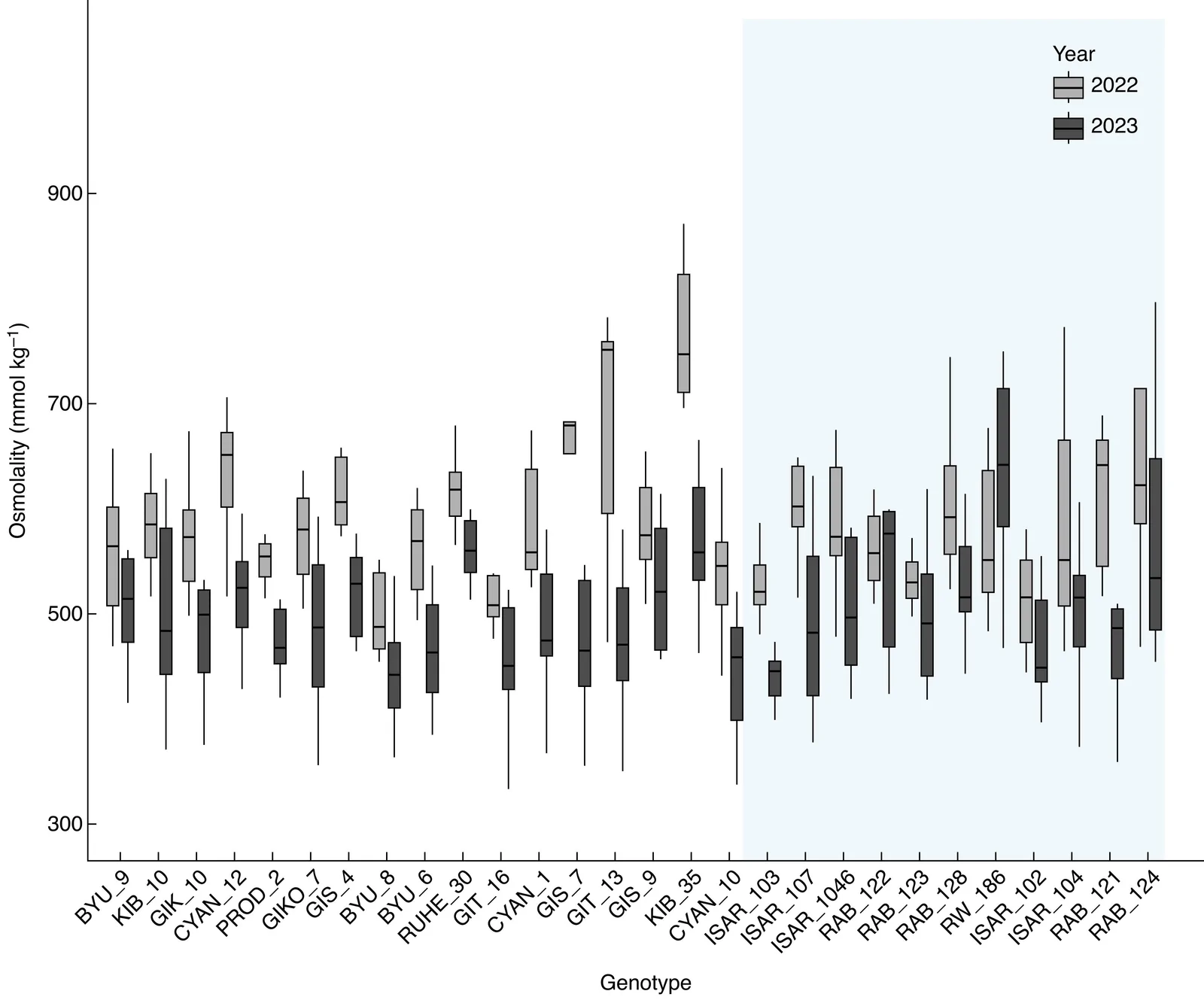
Leaf osmolality. Leaf osmolality (mmol kg^−1^) in irrigated plants across two field seasons in Rwanda. Boxplots show interquartile range (boxes), median (horizontal line) and individual outliers as dots. Light grey: 2022; dark grey: 2023. The light blue shading corresponds to improved cultivars.

Net photosynthesis (*A*_net_) and *g*_s_ were strongly correlated across genotypes (*R*^2^ = 0.71, *P* < 0.001; Fig. 5A). There was no correlation between net photosynthesis and SPAD (Supplementary Data Fig. S3). Finally, iWUE was weakly correlated to iWUE calculated from gas exchange measurement (iWUE_inst_, *R*^2^ = 0.20, *P* = 0.014) as well as both components *g*_s_ (*R*^2^ = 0.22, *P* = 0.01) and *A*_net_ (*R*^2^ = 0.19, *P* = 0.020; Fig. 5B– D).

**Fig. 5.**
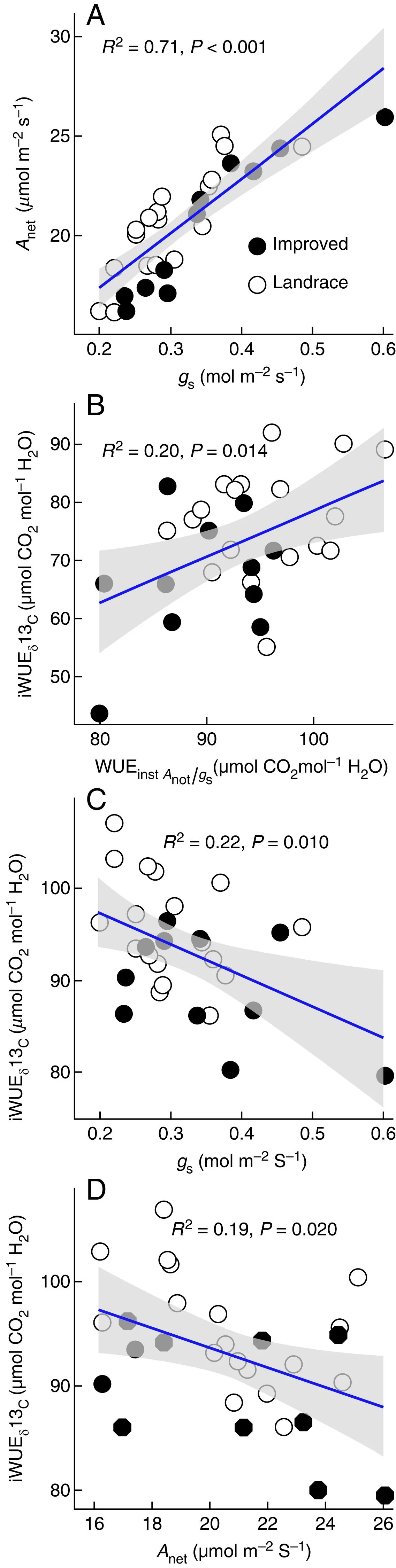
Relationships among leaf-level physiological traits in common bean. Linear regressions of genotype means for key leaf-level traits in common bean. The panels show the relationships between the following variables: (A) photosynthetic rate (*A*_net_, µmol m^−2^ s^−1^) and stomatal conductance (*g*_s_, mol m^−2^ s^−1^); (B) iWUE (µmol CO_2_ mol^−1^ H_2_O) derived from leaf carbon isotope discrimination and iWUE_inst_ (µmol CO_2_ mol^−1^ H_2_O) estimated as the ratio *A*_net_/*g*_s_; (C) intrinsic water-use efficiency (iWUE, µmol CO_2_ mol^−1^ H_2_O) estimated from leaf carbon isotope composition and *g*_s_; and (D) iWUE and *A*_net_. Each point represents a genotype mean; open circles denote landraces and solid black circles denote improved cultivars. Lines represent least-squares fits with 95 % confidence bands. The *P*-value and *R*^2^ for each regression are shown within each panel.

### Drought effects, yield and phenology

Seed yield per plant under irrigation was consistent across years and genotypes (Supplementary Data Fig. S4). Yield under drought was less consistent than under irrigation but was still strongly correlated across years and genotypes. Drought significantly reduced overall seed yield (*F*_(12,069)_ = 13.93, *P* < 0.001, *n* = 2189 plants, after excluding non-germinated and diseased plants), with a stronger effect observed in 2023 (on average a loss of 28 g of seeds per plant, corresponding to a 14.6 % reduction) than in 2022 (average loss 12 g per plant, corresponding to an 8.3 % reduction), consistent with the stronger and more uniform drought treatment in 2023. A similar number of plants died in both treatments and deaths mainly occurred prior to initiation of drought treatment: a total of 212 plants died in 2022 and 115 in 20.

Five cultivars showed significant reductions in seed yield per plant under drought in 2023, whereas only three cultivars demonstrated significant reduction in 2022 (Fig. 6). Two cultivars (KIB_35 and GIS_9) with low yield under both irrigated conditions and drought treatment showed a significant increase in seed yield per plant in response to drought in 2023. This was explained by a significant increase in the number of seeds per plant. However, the cultivar GIS_9 did not show this pattern in the 2022 season, when its yield and number of seeds instead decreased (Fig. 6A). Thirteen cultivars displayed a reduction in specific seed weight in response to the drought treatment in 2023 (Fig. 6B), whereas only three cultivars showed reduction in 2022 (Fig. 6A).

**Fig. 6.**
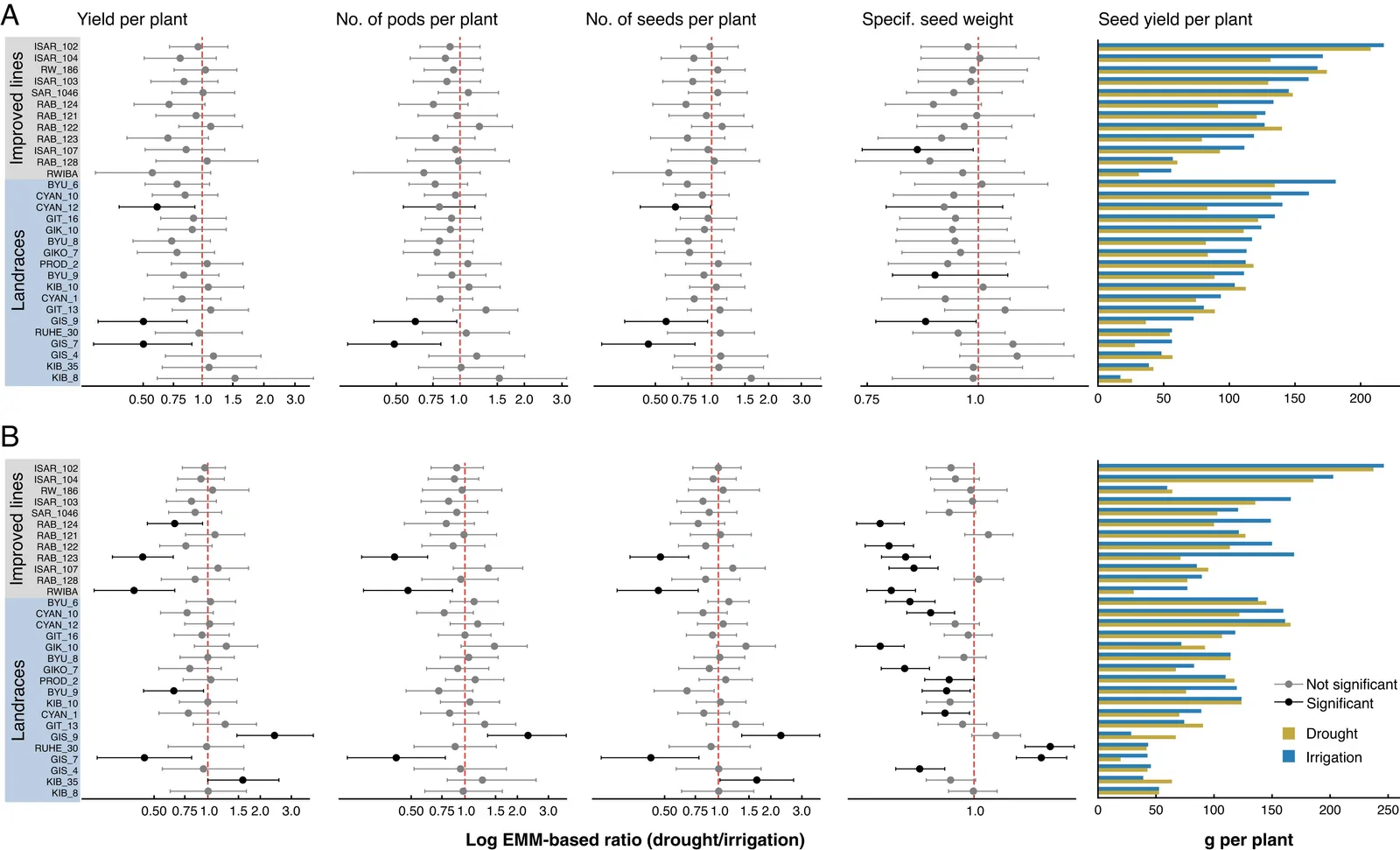
Effects of drought on yield parameters across common bean genotypes. Results of generalized linear mixed models (GLMMs) showing the effect of drought relative to irrigation on seed yield per plant, number of pods per plant, number of seeds per plant and specific seed weight across common bean genotypes. The results are shown for two consecutive field seasons in Rwanda, 2022 (A) and 2023 (B). Symbols represent estimated drought-to-irrigation ratios for each genotype, with horizontal bars indicating 95 % confidence intervals. Open circles denote landraces, and solid black circles denote improved lines. Estimates significantly different from 1.0 (*P* < 0.05) are shown in black, while non-significant estimates are shown in grey.

The number of days to flowering was positively associated with the number of days to maturity across years and water regimes. In 2022, flowering time explained approximately half of the variation in time to maturity under both drought (*R*^2^ = 0.50, *P* < 0.001) and irrigation (*R*^2^ = 0.5, *P* < 0.001). The relationship was stronger in 2023, with flowering time explaining a larger proportion of the variation in maturity under drought (*R*^2^ = 0.68, *P* < 0.001) and irrigation (*R*^2^ = 0.60, *P* < 0.001). Time to maturity (Supplementary Data Fig. S5) decreased by on average 2 d under drought during the 2022 season (β = −2.02 ± 0.92 s.e., *P* = 0.029) and by 3 d in 2023 (β = −2.88 ± 0.99 s.e., *P* = 0.0038). However, the response of time to maturity to drought did not differ significantly among cultivars. Pod harvest index (PHI) varied among cultivars, ranging from approximately 0.7 to 0.8 (Fig. S6), but was not significantly reduced under drought conditions.

### Relationships between yield and other traits

Leaf-level physiological traits were tested against seed yield per plant under irrigated conditions (Fig. 7). Stomatal conductance (*g*_s_; $R^{2}=0.29$, P=0.003; Fig. 7A) and net photosynthesis ($A_{\mathrm{net}}$; $R^{2}=0.24$, P=0.007; Fig. 7B) were positively associated with yield under irrigation. However, the strongest leaf-level predictor of seed yield was iWUE estimated from carbon isotope discrimination (*R*^2^ = 0.48, *P* < 0.001; Fig. 7C). Improved cultivars tended to appear above the regression line for *A*_net_ against seed yield per plant (Fig. 7B), suggesting a more efficient allocation of photosynthates to the seeds in the improved cultivars. Nevertheless, considerable variation among cultivars remained, with several cultivars deviating markedly from the overall relationship between yield and iWUE.

**Fig. 7.**
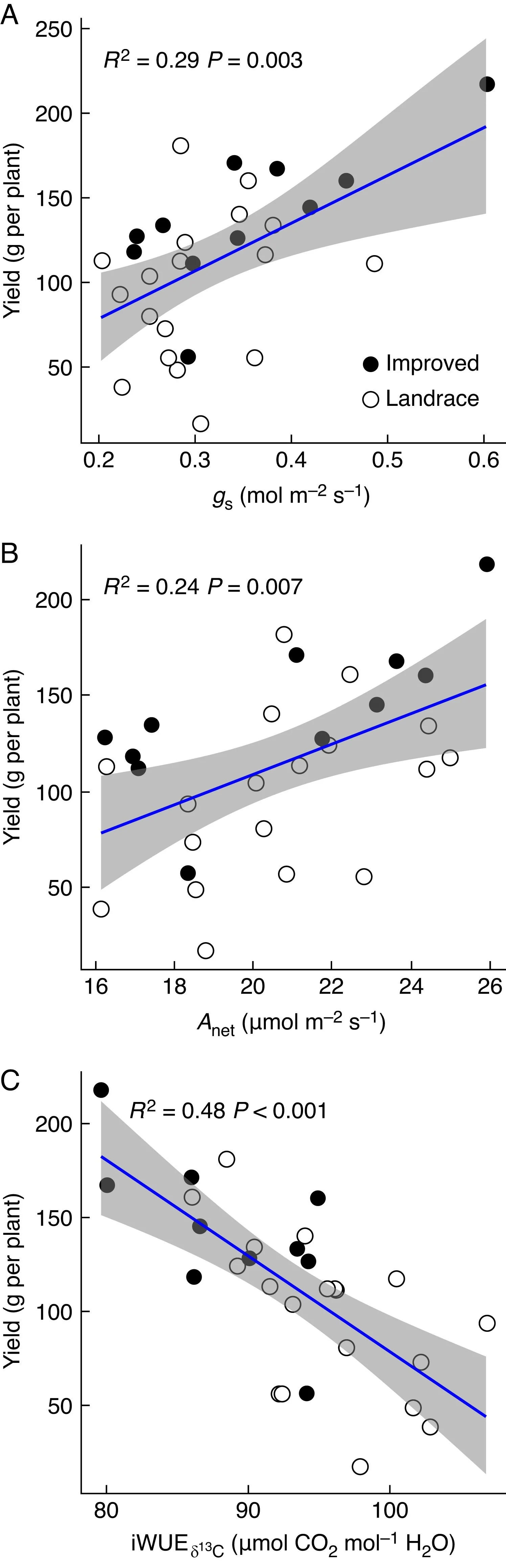
Relationships between yield and leaf-level physiological traits. Linear regressions between cultivar means: (A) yield (g per plant) vs stomatal conductance (*g*_s_, mol m^−2^ s^−1^), (B) yield (g per plant) vs photosynthetic rate (*A*_net_, µmol m^−2^ s^−1^) and (C) yield (g per plant) vs intrinsic water-use efficiency (iWUE, µmol CO_2_ mol^−1^ H_2_O) estimated from leaf carbon isotope composition (δ^13^C). Points are genotype means; open circles denote landraces and filled circles denote improved varieties. Lines show ordinary least-squares fits with 95 % confidence bands; *P*-values and R^2^ are shown in each panel.

Finally, we tested whether leaf-level physiological traits measured prior to the onset of drought treatment could predict cultivar-specific responses to drought, quantified as the change in seed yield between irrigated and drought treatments (Supplementary Data Fig. S7). Yield response todrought was not significantly correlated to any of the measured leaf-level traits (*g*_s_, *A*_net_, iWUE calculated from isotopic discrimination, and leaf osmolality). The yield response to drought was also not significantly correlated to days to maturity nor PHI. However, seed yield per plant increased with days to maturity across both water regimes (Fig. 8A and C). On average, seed yield per plant increased by approximately 4 % per additional day to maturity. A few cultivars deviated markedly from this relationship. In particular, the improved cultivars, ISAR_102 and ISAR_104, displayed disproportionately high yield in relation to their short time to maturity. PHI was positively associated with seed yield under drought in 2023 (*R*^2^ = 0.19, *P* = 0.016; Fig. 8D), whereas the relationship under irrigation was weaker and not statistically significant (*R*^2^ = 0.11, *P* = 0.071 Fig. 8B). These results suggest that cultivars with higher PHI tended to achieve greater yields under water-limited conditions, even though drought did not significantly affect PHI itself.

**Fig. 8.**
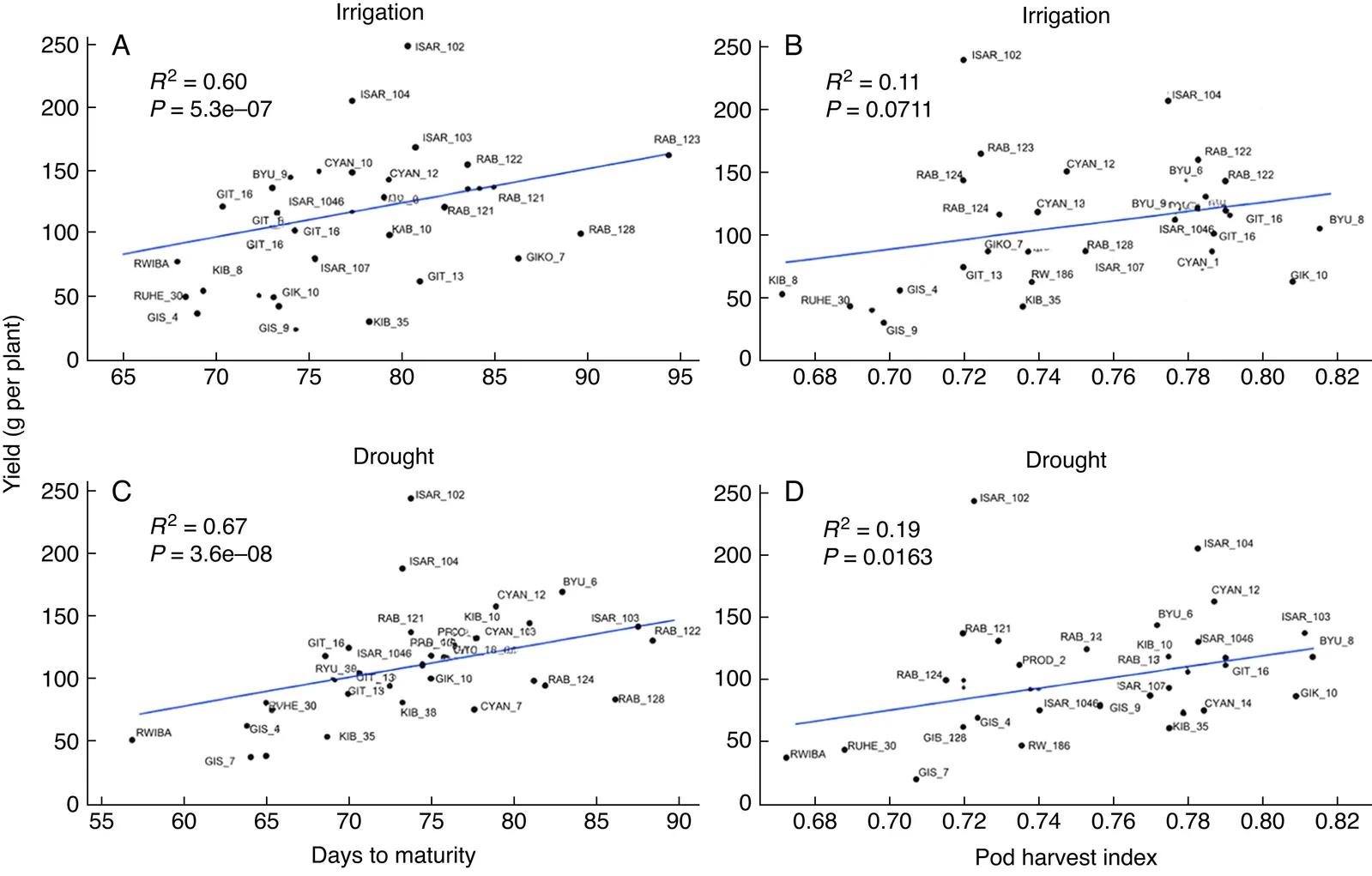
Relationship between seed yield, time to maturity and pod harvest index. Linear relationships between seed yield per cultivar and days to maturity (A and C) or pod harvest index (PHI; B and D) in the 2023 field experiment. Relationships are shown separately for irrigated (A, B) and drought (B, D) treatments. Solid lines indicate fitted linear regressions; corresponding *R*^2^ and *P*-values are shown within each panel.

## DISCUSSION

In this study we evaluated a selection of improved cultivars and landraces of common bean with respect to leaf-level physiological traits and the effect of terminal drought treatment on phenology and yield parameters. Our results reveal a large degree of genotypic variation and strong correlations between yield and low iWUE. Our results have implications for breeding and further studies on the genetics of water-use and drought tolerance traits.

### Genotypic variation in leaf-level physiological traits and correlations with seed production

We found substantial genotypic variation in net photosynthetic rate (*A*_net_) and stomatal conductance (*g*_s_) among cultivars under irrigated conditions at the vegetative stage. This indicates inherent differences in carbon assimilation and water-use regulation among genotypes. As expected, higher *g*_s_ was strongly correlated with higher *A*_net_ (Yamori *et al*., 2020). As a time-integrated measure we analysed iWUE from stable carbon isotopes (δ^13^C) in leaf tissue (Condon *et al*., 2002). This parameter also exhibited substantial genotypic variation. The direct leaf-level measurements of *A*_net_ and *g*_s_ were correlated with iWUE. However, the correlations were not very strong, implying that snapshot measurements do not give the same picture of carbon fixation and water loss over a longer period as the analysis of δ^13^C-iWUE. However, as previously described for genotypic differences in soybean (Gilbert *et al*., 2011), *g*_s_ is the more strongly correlated parameter to iWUE. The speed of stomatal responses to changes in light intensity (Lawson and Vialet-Chabrand, 2019), stomatal density (Bertolini *et al.,* 2019) and changes to the gas exchange parameters over time might add a layer of complexity not reflected in snapshot measurements. Nevertheless, a more complete picture of underlying processes is gained from combining isotope analysis and gas exchange measurements.

Seed yield per plant showed a strong correlation to all three measured traits: *A*_net_, *g*_s_ and iWUE. This is again an intuitive correlation as *g*_s_ would allow a high rate of photosynthesis, which in its turn is a prerequisite for high seed production. This has also been established previously for several crops (Roche, 2015). Breeding for high productivity has been indirectly driving development of crops with high transpiration rates (Blum, 2009). While this trend holds true for the cultivars of common beans in our study, there are some important exceptions to the trend that should be noted. The improved cultivars tended to exhibit higher yield per unit of *A*_net_ compared to landraces, demonstrating that a more efficient conversion of assimilated carbon into reproductive biomass has been achieved in these cultivars. In contrast, several landraces combined lower *g*_s_ and *A*_net_ with moderate iWUE values, reflecting more conservative water-use strategies that may favour survival over maximum productivity. Additionally, while there is a strong correlation between low iWUE and high seed production, there is considerable variation between cultivars, with some being more efficient than expected. There was a large overlap between improved cultivars and landraces. Thus, in the case of the climbing cultivars of common bean that were included in this study under realistic field conditions, breeding has not led to a very strong selection for lower iWUE. These results demonstrate that, in common bean, the assumption that higher productivity necessarily requires greater water consumption does not necessarily hold true and that underlying genetic components might be amenable for further mapping and breeding.

### Effects of drought treatment and relationship to leaf-level physiological traits

Yield under irrigated conditions was highly consistent between 2022 and 2023, indicating strong environmental and genotypic stability in the absence of stress. The drought treatment was overall more severe in 2023, as indicated by soil moisture reduction, whereas rain toward the end of the experimental period in 2022 resulted in a shorter and a more intermittent drought treatment in 2022. These differences might explain the different responses on a genotype basis between the years. Specific seed weight decreased significantly in response to drought for most cultivars in the 2023 season, indicating that the drought treatment had a significant impact on the plants. Achieving a sufficient level of drought stress under field conditions and at the same time managing the risk of extreme conditions that lead to such strong reductions in yield that the genotypic variation disappears is clearly challenging (Beebe *et al*., 2013). Including additional levels of drought, or drought stress initiation at different stages in the life cycle could be helpful in managing this trade-off. In this study, however, we prioritized the inclusion of more cultivars over additional treatment levels, to enable screening of a large set of genotypes for variation in physiological traits. Our study clearly shows differential sensitivity among cultivars to drought during the pod filling stage, which is a common type of stress exposure in Rwandan agriculture where the maturity of bean crops tends to co-occur with the onset of the dry season (Katungi *et al*., 2018).

Time to maturity was shortened by 2 d on average under drought, consistent with a drought-escape response (Geleta *et al*., 2024). This phenological adjustment appears to be a reliable and reproducible mechanism of drought adaptation in common bean (Beebe *et al*., 2013). We could, however, not reliably establish genotypic differences in this response. While drought escape can be an effective strategy, it entails potential trade-offs, as increased earliness often comes at the expense of reduced yield potential (White and Singh, 1991). Another previously observed pattern is that high-yielding common bean cultivars may maintain superior yield under drought, despite absolute reductions in yield (Acosta-Díaz *et al*., 2004). This was also evident in our study (Fig. 6): although the yield of several improved cultivars declined under drought, their absolute yield remained higher than that of other cultivars that were less affected by the drought treatment.

While the drought treatment caused an overall reduction in time to maturity in our experiment, there was a general positive relationship between time to maturity and yield in both irrigated and drought conditions. A longer time to maturity appears to allow more photosynthate to partition into the seeds. In this respect, however, there were also some notable outlier cultivars with much higher seed yield in a shorter time than would be expected based on the general trend in the dataset. High productivity within a short time would be a highly valuable trait when plants are exposed to drought at late developmental stages and could be considered a form of drought avoidance.

PHI remained relatively stable across treatments and years (Supplementary Data Fig. S5) Although PHI is recognized as an important trait for drought tolerance in common bean (Polania *et al*., 2016), the absence of a strong drought effect in this study may suggest that the stress intensity was insufficient to substantially impair partitioning efficiency, or that the evaluated germplasm maintained a relatively stable reproductive allocation under stress. Nevertheless, there was a weak correlation between PHI and yield under drought in our experiment, consistent with previous observations for common bean (Polania *et al*., 2016) and indicating that high PHI may be a useful trait for improved productivity under drought.

Despite strong correlations with yield in both irrigated and drought conditions, neither δ^13^C-derived iWUE nor *g*_s_ explained the effects of drought on seed yield in our experiment. This supports the argument that high iWUE should not be equated with drought resistance, because high WUE is often achieved through reduced water use rather than increased productivity, favouring conservative traits that limit yield potential (Blum, 2009). The potential benefits of improved water economy are not captured in this specific experiment, where the cultivars were mixed rather than grown in separate blocks where the conservative genotypes would experience slower soil water reduction than the big spenders (Condon *et al*., 2004). The lack of clear relationships between leaf-level physiological traits and yield loss under drought highlights the multifactorial nature of the effect of drought on yield. Many additional factors contribute to productivity under drought stress, such as disease resistance and root architecture (Beebe *et al*., 2013), none of which could be included in this experiment. The complexity of yield responses to drought accentuates the need for more knowledge about the underlying physiological responses and the contribution of different factors.

Baseline leaf-level osmolality in unstressed plants and the drought response of yield showed no significant relationship in this study. This contrasts with the identification of leaf osmotic potential in unstressed plants as a good predictor for drought tolerance on a broader ecological scale (Bartlett *et al*., 2012*b*). Osmotic adjustment is recognized as an important drought tolerance mechanism that allows plants to maintain turgor and metabolic functions under low water potential (Sassi *et al*., 2010; Bartlett *et al*., 2012*b*). Only a few studies have looked at leaf osmolality and osmotic adjustment in common bean and these report conflicting results regarding the relative importance of osmotic adjustment or changes in cell wall elasticity (Martínez *et al*., 2007; Lanna *et al*., 2016). In our experiments, it was also clear that leaf osmolality was more sensitive to environmental variation than the other physiological traits that were studied. Leaf osmolality showed lower stability across years and was not correlated to any other physiological parameters or with yield. In our study, the soil water content in 2022 was lower at the beginning of the growing season than in 2023. This might explain the pattern of higher leaf osmolality in 2022 than in 2023. Further and more controlled studies are needed to better understand the response of leaf osmolality to drought stress and the potential genotypic differences for this trait in common bean. However, it is clear that the osmolality of unstressed leaves is not a good predictor for drought tolerance in common bean.

### Conclusions and perspectives for breeding and genetic mapping

Physiological traits provide multiple paths for improving water use and productivity under water-limited conditions in common bean. Although landraces were not consistently more drought-resistant than improved cultivars, some showed remarkable resilience to drought. Conversely, some improved cultivars maintained the highest yields overall, confirming that strong yield potential may buffer productivity losses under drought stress. The substantial genotypic variation observed among both improved cultivars and landraces underscores the opportunity to combine high yield potential with adaptive physiological traits. In a breeding perspective, it might also be a plausible way forward to use a few genotypes with reasonable yield but still relatively good water use properties combined with low sensitivity to drought. In our experiment, the improved variety ISAR_103 and the landraces Byumba 6 and Gitarama 16 stand out as combining fair yield with low water use and low sensitivity to drought. Crucially, our results indicate that traits that would contribute to more efficient use of water, allocation of carbon into developing seeds and tolerance of low soil water content are separable and that the underlying genetic variation could potentially be understood and used for breeding.

## Supplementary Material

mcag257_Supplementary_Data

## Data Availability

All the raw data are available in the Supplementary Data Table S1.

## SUPPLEMENTARY DATA

Supplementary data are available at *Annals of Botany* online and consist of the following. Figure S1: Experimental layout. Eight plots (A–H) were arranged along the field. Each plot measured 10 m × 8 m and plots were separated by 2-m alleys. Within each plot, rows were spaced 1 m apart with 0.5-m within-row plant spacing and 0.5-m border margins on all sides. Red dots indicate the position of access tubes that were used for the soil moisture measurements. Figure S2: Temperature and humidity during the two experimental seasons in Rwanda. Daytime (black) and night-time (grey) relative humidity (%; top panels) and air temperature (°C; bottom panels) were recorded continuously at 1.5 m height above ground using Tinytag loggers throughout the 2022 (A) and 2023 (B) dry-season field experiments. Data represent daily means. Figure S3: Correlation between SPAD and CO_2_ assimilation rate. Relationship between cultivar mean chlorophyll content (SPAD) and net photosynthesis (*A*_net_) measured under well-watered conditions prior to drought imposition. Filled circles represent improved cultivars and open circles represent landraces. The blue line shows the fitted linear regression and the shaded area indicates the 95 % confidence interval. No significant association was observed. Figure S4: Correlation between seed yield per plant across two field seasons. Relationship between mean yield (g per plant) in 2022 and 2023 under (A) irrigation and (B) drought conditions across common bean genotypes. Each point represents the mean of a genotype; white circles with black outline indicate landraces, and solid black circles indicate improved varieties. Regression lines (±95 % CI) are shown, with *R*^2^ and *P*-values indicated in each panel. Figure S5: Days to maturity. Days to maturity recorded in 2022 and 2023. Blue boxes represent irrigated plants, red drought treated and the light grey area denotes improved cultivars. Figure S6: Pod harvest index. Pod harvest index (g g^−1^) in 2022 and 2023 was recorded as the weight of seeds per plant divided by the sum of seed and husk weight. Blue boxes represent irrigated plants, red drought treated and the light grey area denotes improved cultivars. Figure S7: Relationships between leaf-level physiological traits and effect of drought treatment on yield in common bean. Associations between physiological traits and drought tolerance across genotypes, expressed as the drought-to-irrigation yield ratio (D/W) derived from model-estimated genotype means. Panels A–D show models fitted to 2022 data: (A) intrinsic water-use efficiency (‰), (B) leaf osmolality (mmol kg^−1^), (C) stomatal conductance *g*_s_ (mol m^−2^ s^−1^), and (D) net photosynthesis *A*_net_ (µmol m^−2^ s^−1^). Panels E–F show the same relationships for 2023: (E) intrinsic water-use efficiency and (F) osmolality. Each point represents the mean value of a genotype; black symbols indicate genotypes with a significant drought effect, while grey symbols indicate non-significant. Solid black lines are ordinary least-squares fits with shaded 95 % confidence bands; the dashed horizontal line denotes D/W = 1 (no drought effect). The coefficient of determination (*R*^2^) and *P*-value for each regression are reported within panels; a subset of genotypes that showed a significant drought response is labelled for reference.
