# Supplementary material for: Genotypic variation in water-use traits and drought response of Rwandan common bean cultivars
Source: Ann Bot. 2026 Aug 17;138(5):2029–44. doi: 10.1093/aob/mcag257 (PMC13715028; doi:10.1093/aob/mcag257)
Supplement: Supplementary file 1 [file mcag257_supplementary_data.zip › Supplemental figures 1-7.pdf]

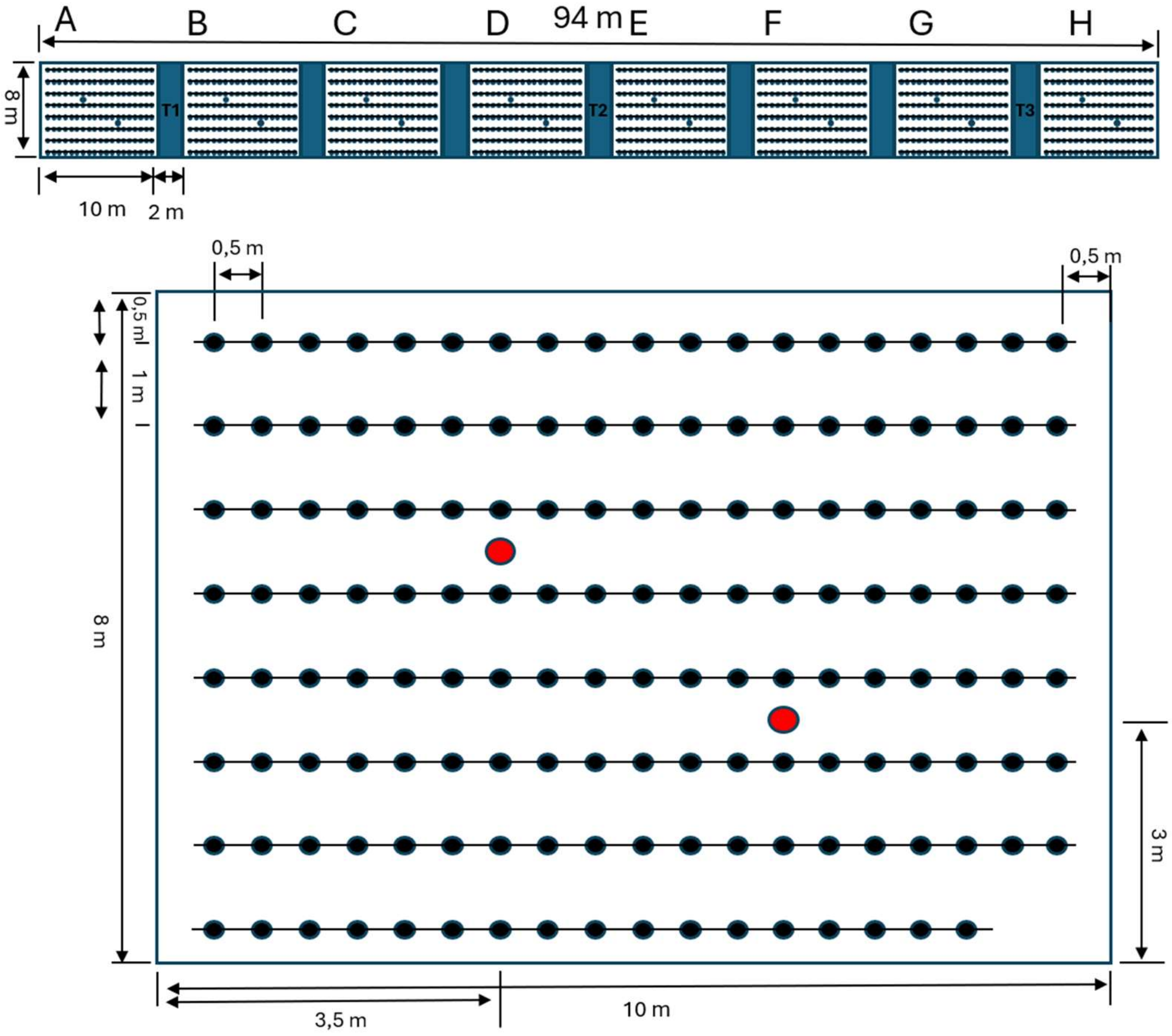

Figure S1. Experimental layout

Eight plots (A–H) were arranged along the field. Each plot measured 10 m × 8 m and plots were separated by 2 m alleys. Within each plot, rows were spaced 1 m apart with 0.5 m within-row plant spacing and 0.5 m border margins on all sides. Red dots indicate the position of access tubes that were used for the soil moisture measurements.

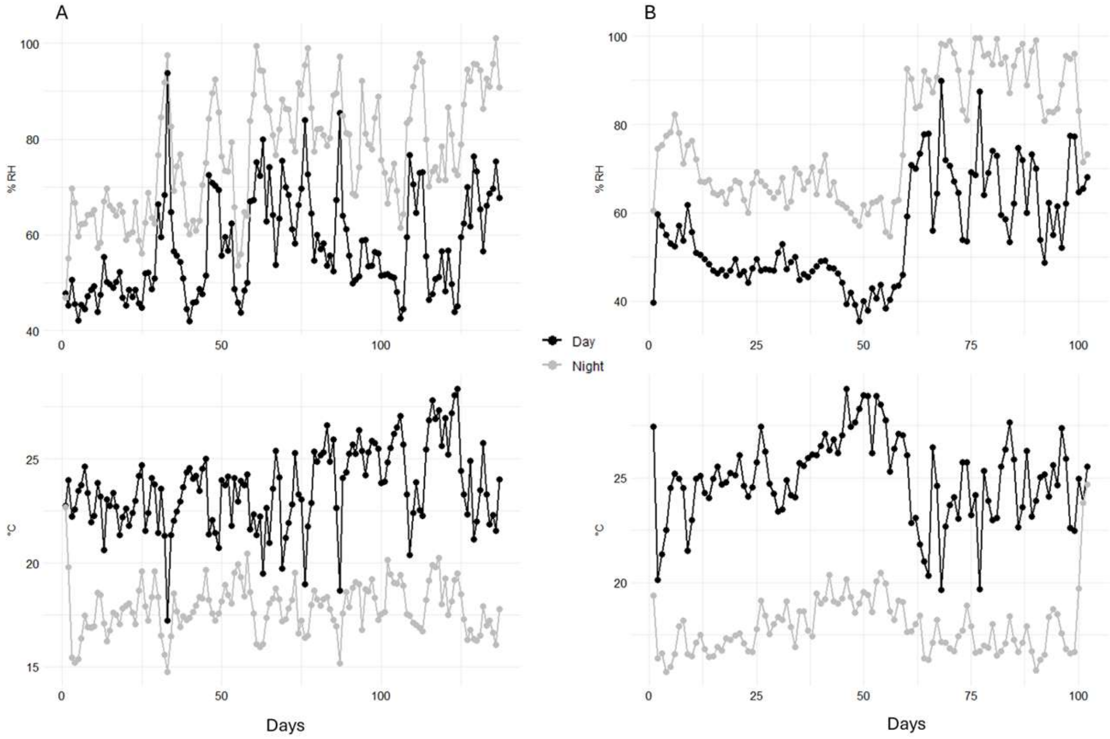

Figure S2. **Temperature and humidity during the two experimental seasons in Rwanda** Daytime (black) and nighttime (grey) relative humidity (%; top panels) and air temperature (°C; bottom panels) were recorded continuously at 1.5 m height above ground using Tinytag loggers throughout the 2022 (A) and 2023 (B) dry-season field experiments. Data represents daily means.

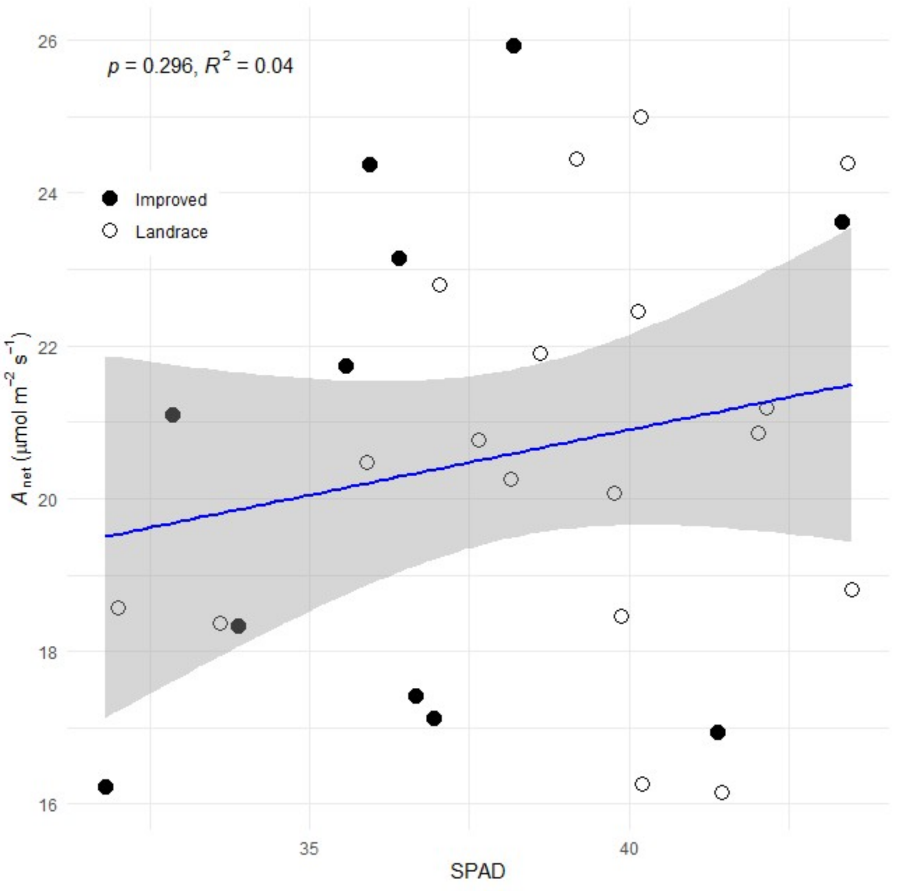

Figure S3. Correlation between SPAD and CO_2 assimilation rate

Relationship between cultivar mean chlorophyll content (SPAD) and net photosynthesis (A_{net}) measured under well-watered conditions prior to drought imposition. Filled circles represent improved cultivars and open circles represent landraces. The blue line shows the fitted linear regression and the shaded area indicates the 95% confidence interval. No significant association was observed.

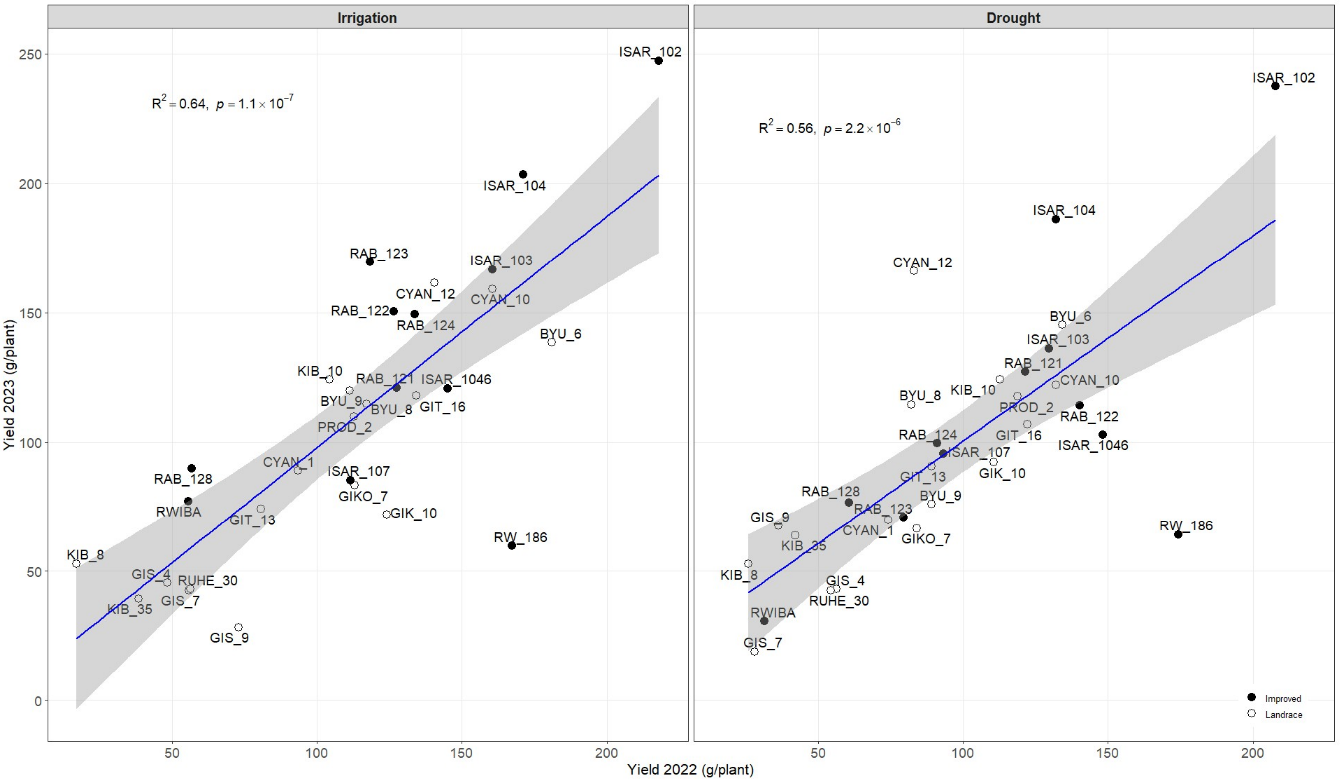

Figure S4. Correlation between seed yield per plant across two field seasons
Relationship between mean yield (g/plant) in 2022 and 2023 under (A) irrigation and (B) drought conditions across common bean genotypes. Each point represents the mean of a genotype; white circles with black outline indicate landraces, and solid black circles indicate improved varieties. Regression lines (±95% CI) are shown, with R^2 and *p*-values indicated in each panel.

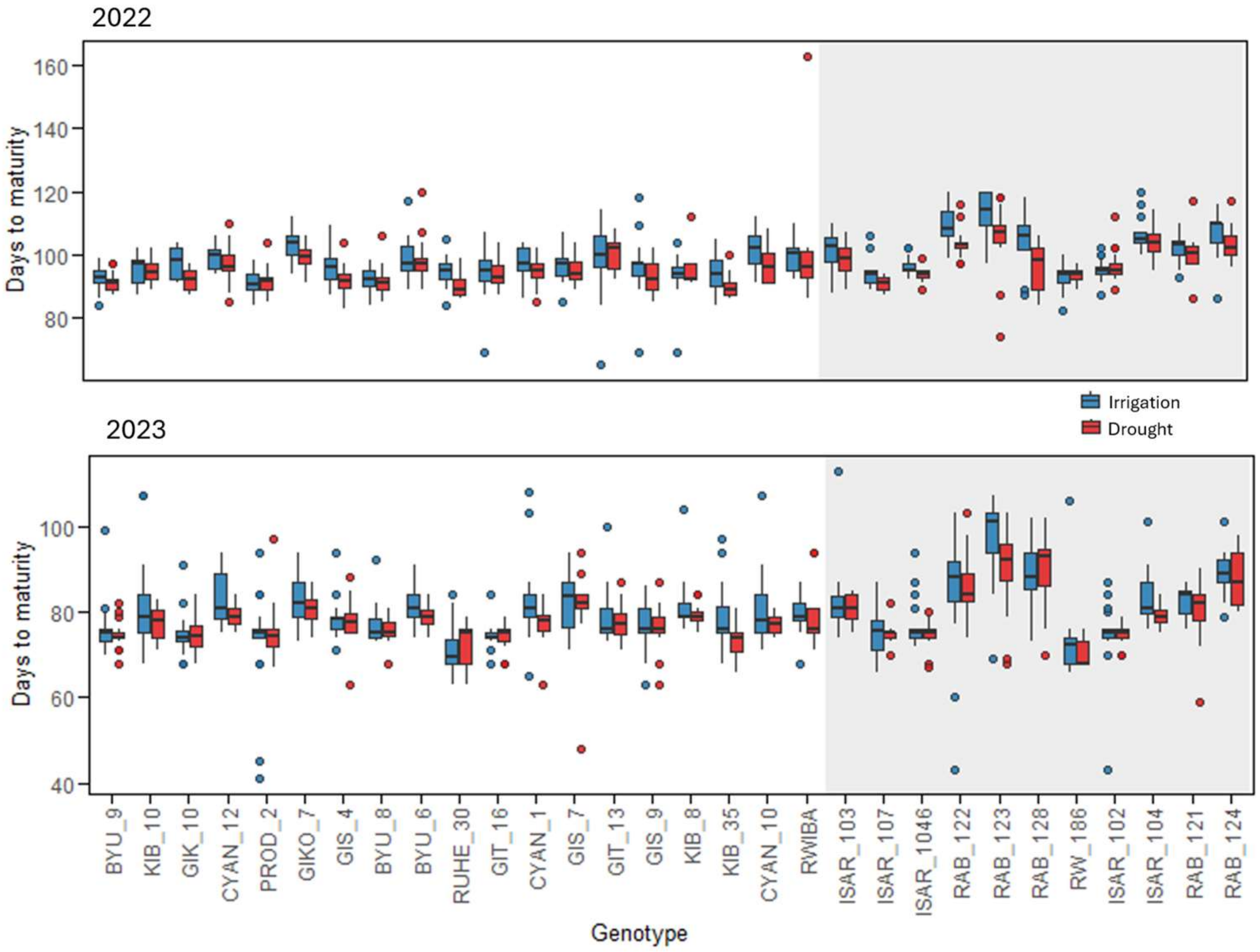

Figure S5. Days to maturity

Days to maturity recorded in 2022 and 2023. Blue boxes represent irrigated plants, red drought treated, and the light grey area denotes improved cultivars.

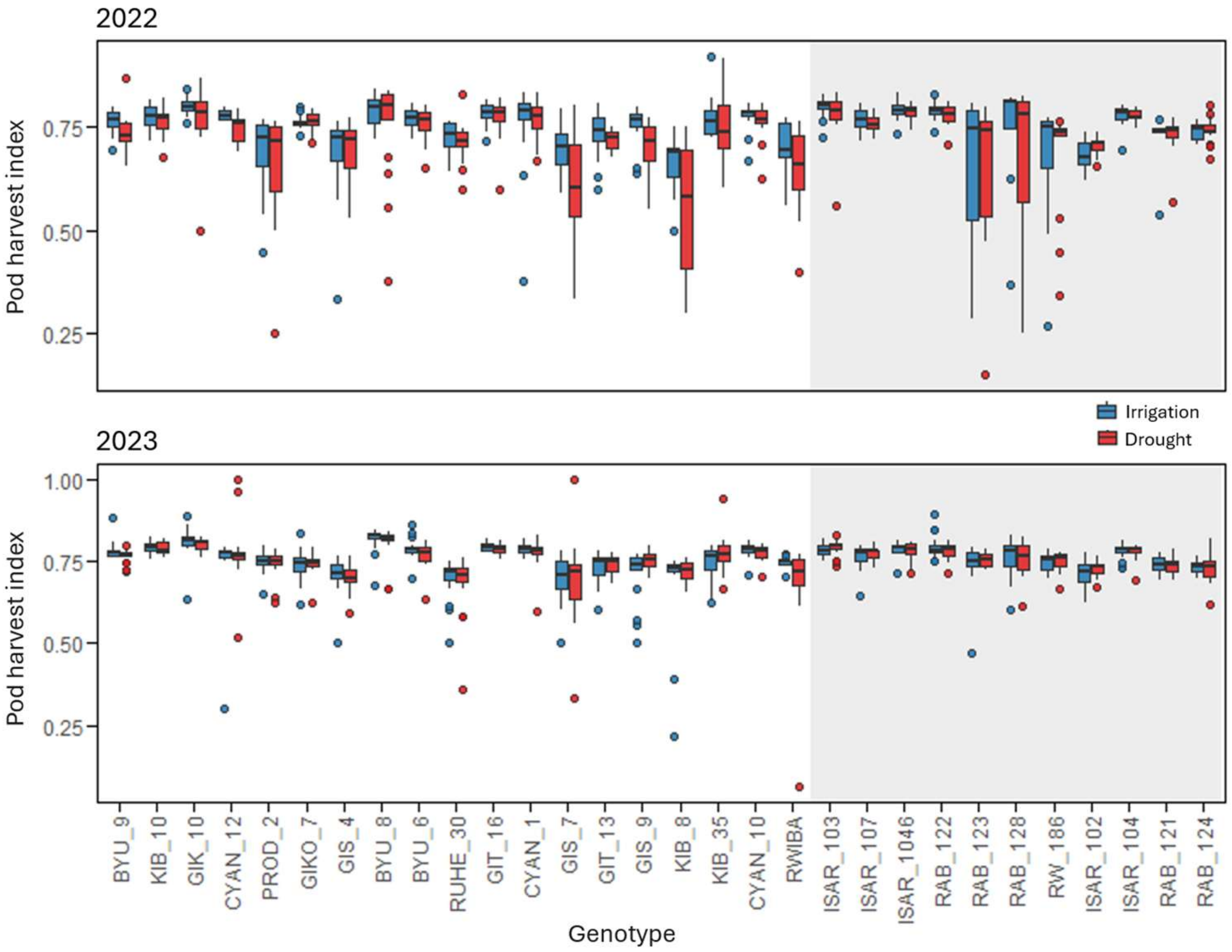

Figure S6. Pod harvest index

Pod harvest index ($g*g^{-1}$) in 2022 and 2023 was recorded as the weight of seeds per plant divided by the sum of seed and husk weight. Blue boxes represent irrigated plants, red drought treated, and the light grey area denotes improved cultivars.

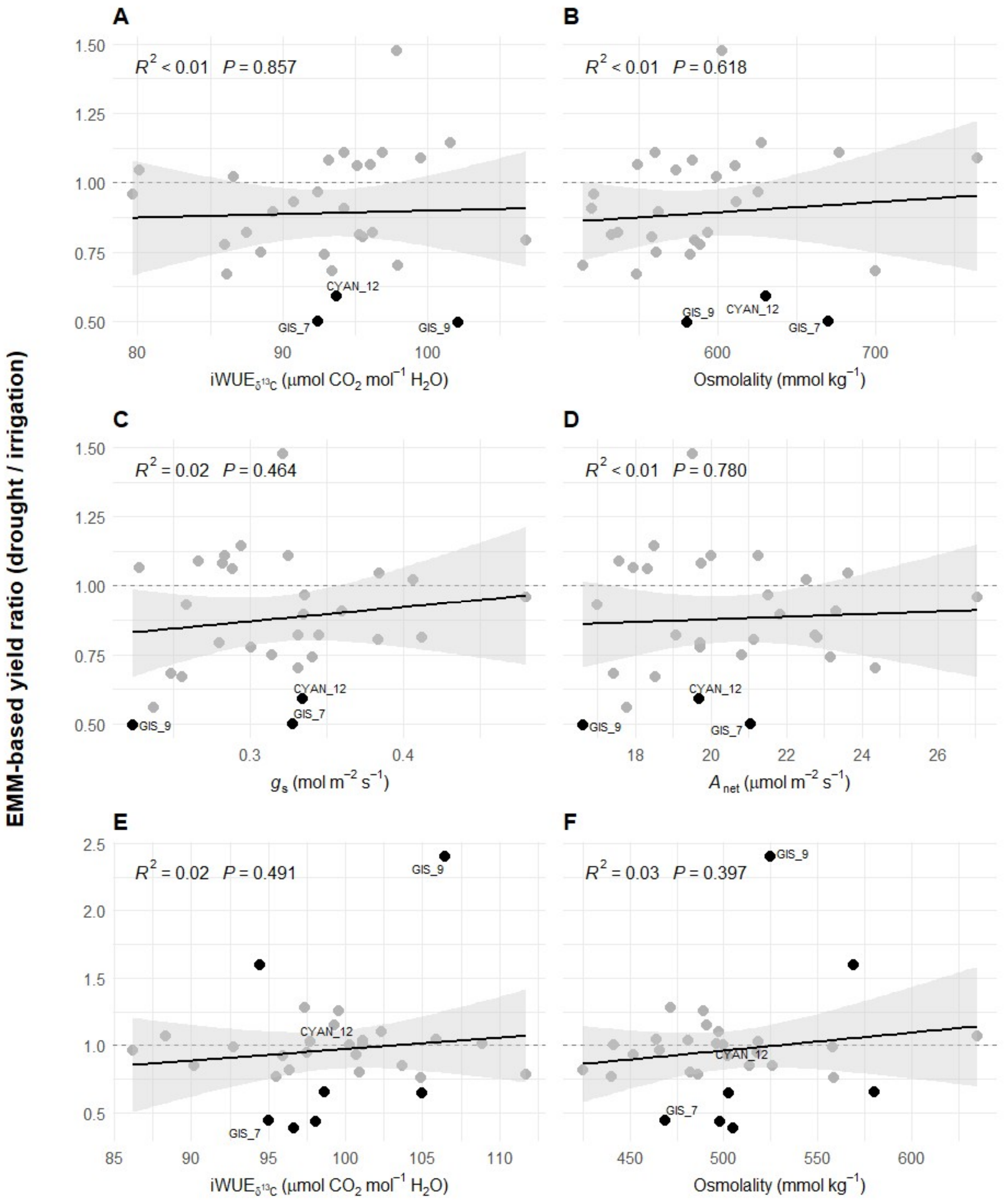

Figure S7. Relationships between leaf-level physiological traits and effect of drought treatment on yield in common bean

Associations between physiological traits and drought tolerance across genotypes, expressed as the drought-to-irrigation yield ratio (D/W) derived from model-estimated genotype means. Panels A–D show models fitted to 2022 data: (A) intrinsic water-use efficiency (‰), (B) leaf osmolality (mmol kg^{-1}), (C) stomatal conductance g_s (mol m^{-2} s^{-1}), and (D) net photosynthesis A_{net} (µmol m^{-2} s^{-1}). Panels E–F show the same relationships for 2023: (E) intrinsic water-use efficiency and (F) osmolality. Each point represents the mean value of a genotype; black symbols indicate genotypes with a significant drought effect, while grey symbols indicate non-significant. Solid black lines are ordinary least-squares fits with shaded 95% confidence bands; the dashed horizontal line denotes D/W = 1 (no drought effect). The coefficient of determination (R^2) and p-value for each regression are reported within panels; a subset of genotypes that showed a significant drought response is labeled for reference.
